# Hodgkin Lymphoma: A Special Microenvironment

**DOI:** 10.3390/jcm10204665

**Published:** 2021-10-12

**Authors:** Giuseppina Opinto, Claudio Agostinelli, Sabino Ciavarella, Attilio Guarini, Eugenio Maiorano, Giuseppe Ingravallo

**Affiliations:** 1 Haematology and Cell Therapy Unit, IRCCS-Istituto Tumori ‘Giovanni Paolo II’, 70124 Bari, Italy; g.opinto@oncologico.bari.it (G.O.); s.ciavarella@oncologico.bari.it (S.C.); attilio.guarini@oncologico.bari.it (A.G.); 2 Haematopathology Unit, IRCCS Azienda Ospedaliero-Universitaria di Bologna, 40138 Bologna, Italy; claudio.agostinelli@unibo.it; 3 Department of Experimental, Diagnostic and Specialty Medicine (DIMES), University of Bologna, 40138 Bologna, Italy; 4 Section of Pathology, Department of Emergency and Organ Transplantation (DETO), University of Bari Aldo Moro, 70124 Bari, Italy; eugenio.maiorano@uniba.it

**Keywords:** Classical Hodgkin’s lymphoma, microenvironment, gene expression profiling, therapy

## Abstract

Classical Hodgkin’s lymphoma (cHL) is one of the most particular lymphomas for the few tumor cells surrounded by an inflammatory microenvironment. Reed-Sternberg (RS) and Hodgkin (H) cells reprogram and evade antitumor mechanisms of the normal cells present in the microenvironment. The cells of microenvironment are essential for growth and survival of the RS/H cells and are recruited through the effect of cytokines/chemokines. We summarize recent advances in gene expression profiling (GEP) analysis applied to study microenvironment component in cHL. We also describe the main therapies that target not only the neoplastic cells but also the cellular components of the background.

## 1. Classical Hodgkin’s Lymphoma

Classical Hodgkn’s lymphoma (cHL) accounts for about 10% of all annual lymphoma diagnosis worldwide. cHL is one of the most singular lymphomas for the presence of large multi-and mononucleated cells named Reed-Sternberg (RS) and Hodgkin (H) cells respectively, admixed with an inflammatory background. Four histological subtypes of cHL are recognized: nodular sclerosis (60–70%), mixed cellularity (20–25%), lymphocyte rich (5%) and lymphocyte-depleted (<5%) subtypes [1]. RS and H detection in an appropriate microenvironment (ME) is the key of cHL diagnosis [2]. 

Rengstl et. al. [3], by filming Hodgkin cell lines in real-time by long-term time-lapse microscopy, suggested that RS cells generates from mitosis and incomplete cytokinesis of H cells followed by subsequent re-fusion of identical mononuclear daughter cells [3].

Microdissection experiments have revealed that RS and H cells carry clonal Ig rearrangements and Ig genes somatic hyper mutations [4], suggesting their origin from a pre-apoptotic germinal center (GC) B cell [5,6]. The expression of the tumor necrosis factor receptor family (TNFR) CD30 is a typical feature of cHL tumoral cells [7]. However, RS cells have an unusual immunophenotype characterized by absence of B cell markers, associated to possible coexpression of molecules of various hematopoietic lineage. [7]

The early event that produces this reprogramming is unknown; several studies reported the hypermethylation of the promoter regions of the transcription factors PU.1, BOB.1/OBF.1, that led to their down regulation [8]. Oct-2, BOB.1/OBF.1 and PU.1 regulate the expression of several genes like CD20 [9], BCL-2 [10], CD19 [11] and CD79A, [12] and furthermore they activate Ig genes transcription. This latter function explains the low level of Ig transcripts in cHL neoplastic cells [13]. Other characteristic B cell transcriptional factors are expressed at low levels (EBF) or inactivated (E2A) by the aberrant expression of competitive inhibitors as Notch-1, Id2 and ABF1 [14,15]. An essential mark of the B cell differentiation of the tumoral clone is the expression of the transcription factor PAX5, although many of its target genes are downregulated [16]. 

RS and H cells are also characterized by the constitutive activation of both the canonical and non-canonical NF-κB signaling pathways [17]. This permanent stimulation is due to the complex network of paracrine and direct interactions between neoplastic cells and microenvironment, mediated by CD30, CD40, BCMA and other surface receptors [18,19,20,21], to recurrent somatic genetic lesions inactivating mutations of negative regulators of NF-κB like TNFAIP3 and NFKBIA in about 40% and 20% respectively [22,23,24], or to copy number gains of genes encoding positive regulators like REL and MAP3K14 in about 40% and 30% of cHL [25,26]. Finally, the latent membrane protein 1 encoded by Epstein-Barr virus, present in RS and H cells of 40% of cHL cases in western country, causes NF-κB activation by mimicking an active CD40 [17].

cHL is also characterized by Jak/Stat pathway activation, principally caused by autocrine and/or paracrine signaling events via interleukin receptors and receptor tyrosine kinases [27,28,29,30], PI3K-AKT and MAPK/ERK pathways [31,32,33,34].

## 2. The Main Component of Tumor Microenvironment (TME)

**Figure 1:**
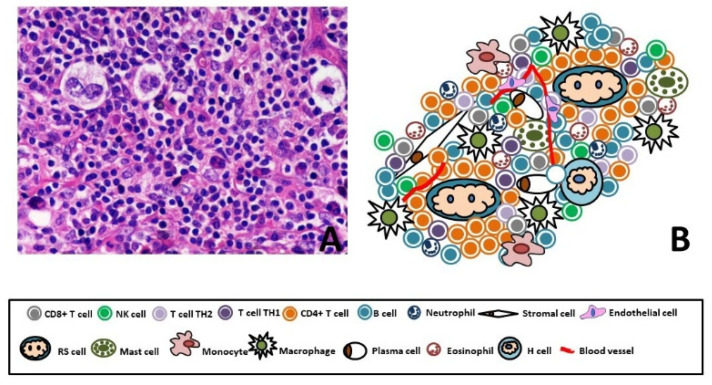
(**A**) Representative figure of Hodgkin Lymphoma (40X, Hematoxylin Eosin). (**B**) Cartoon of Hodgkin Lymphoma, few RS and H cells (1–10%) immerse in a special microenvironment. The 90% of the tumor mass is composed by CD4^+^ Th cells, CD4^+^ Tregs, CD8 T cells, B lymphocytes, plasma cells, histiocytes/macrophages, granulocytes, eosinophils, mast cells, MSCs and endothelial cells.

RS and H cells represent just 1% to 10% of total tumor mass; the remaining 99–90% of the cellular infiltrate is formed by a TME consisting of non-malignant T and B lymphocytes, plasma cells, histiocytes/macrophages, granulocytes, eosinophils, mast cells, mesenchymal stromal cells (MSCs) and endothelial cells [35] (Figure 1).

TME is essential for growth and survival of neoplastic cells and when they metastasize into non-lymphoid organs, they reproduce their cellular background [36]. The TME construction depends on the effect of cytokines/chemokines, including IL-5, IL-7, IL-8, CCL5 (RANTES), CCL17 (TARC), CCL20 and CCL28 produced by RS, H cells and the recruited immune cells [37]. In detail, IL-5, CCL5, CCL28 and the granulocyte-macrophage colony-stimulating factor (GM-CSF) attract the eosinophils [36,38,39], IL-8 is chemotactic for neutrophils [36]; IL-7 increases the proliferation of regulatory T cells [40], CCL5 recruits T-cells, eosinophils and mast cells [18,41], whereas CCL17 and CCL20 attract the T helper (Th) 2 cells and the regulatory T cells [36,42]. Other cytokines and in particular IL-3, IL-4, IL-6, IL-13, IL-15, TGFβ, BAFF, APRIL, RANKL influence the survival of the same tumor cells [36,43]. The dynamics of the TME are also conditioned by cytokines produced by the immune infiltrates at the tumoral site: T cells secrete IL-3, IL-10 and RANKL. IL-3 influences the formation of the inflammatory infiltrate and support the neoplastic cell growth [44], IL-10 promotes strong anti-inflammatory properties [45] and RANKL contributes to activation and survival of dendritic cells [46]. Stromal cells may secrete CCL11 that attracts eosinophils and Th2 cells, IL-7 and CCL5 involved in the growth or survival of RS cells, while dendritic cells may liberate TARCs involved in the recruitment of Th2 cells and regulatory T cells [37]. Macrophages have been shown to secrete cytokines involved in tumor progression such as macrophage migration inhibitory factor (MIF), IL-8 and TNFα. MIF may contribute to the proliferation of RS cells in TME [47,48] and IL-8 may increase neutrophilic infiltration [49,50]. Moreover, mast cell may increase survival signals of tumor cells through CD30L [51].

### 2.1. T Cell Subsets

T cells are the main component of the cHL tumor TME. Several subsets were recognized: CD4^+^ Th cells, CD4^+^ T-regulatory (Tregs) and CD8 cytotoxic T lymphocyte (CTL) [52]. CD4^+^ T cells sometimes are in close contact with RS cells as they can form rosettes around the neoplastic blasts [53,54]. A recent study has been described rosette formation and T cell activation in cHL, using coculture model. In the immune synapse model between RS cells and CD4^+^ T cells, the two interactions TCR-MHCII and CD2-CD58 were needed for T cell activations, while CD2-C58 axis was associated with cell adhesion and rosette formation [55]. 

Previous studies have suggested that the prevalent Th phenotype present in cHL TME is the Th2 [56,57]. This T cell subset is physiologically involved in the eradication of extracellular parasites, but in a tumoral background seems to contribute to the tumor growth [58]. The Th1 subpopulation, is indeed important in host defense against intracellular pathogens and is an effective mediator of anti-tumor immunity [58], seem to be reduced in HL TME. However, a recent study found predominance of an activated, proliferative, and pro-inflammatory cytokine-secretory phenotype, typically of Th1 cells [59]. Normally, CD4^+^ Th cells do not directly demolish malignant tumor cells, but they support the development of tumor immunity by recognizing tumor antigen peptides presented by MHC class II molecules and amplifying the activation and clonal expansion of CTL [60]. In general, CD8^+^ CTL or Natural Killer cells (NK) are the most important effectors of antitumor immunity. The CD8^+^ T cells are a proportion of T-cell infiltrate of Hodgkin lymphoma and are not in close contact with the tumor cells [61]. However paradoxically, an increased numbers of cytotoxic T cells positive for cytotoxic granule-associated RNA-binding protein (TIA1) in the TME correlated with poor outcomes [62,63].

In the TME there is also an accumulation of Treg [60], expressing factor forkhead box P3 (FOXP3) [64]. Treg cells play important roles in limiting immune response and their dysfunction was reported in autoimmune disease [65]. They act their immunomodulatory function through several mechanisms, including overexpression of CTLA4, consumption of IL-2, secretion of IL-10, TGF-β, IL-35 and galectin-1 [66,67,68]. Moreover, Treg cells may facilitate tumor spread, probably inhibiting the antitumor immunity [65]. In several localized or metastatic human carcinomas high infiltration of Treg cells FOXP3^+^ was associated with an unfavorable outcome [69,70,71,72,73,74]. However, in cHL patients, higher number of intra-tumoral FOXP3^+^ Treg cells was, associated with longer DFS and OS, even in multivariate analyses [60]. 

### 2.2. B Lymphocytes

Large quantities of non-malignant B cells are present in the microenvironment of HL but their role in TME is still not well established [75]. Dates of gene profiling provide the association of high intratumoral B-cell counts with better outcome in patients with cHL [76]. Some authors reported that antitumor immunity is highly reduced in the presence of B cells due to down-regulation of both innate and adaptive immunity, in cases where CD40L is expressed by tumor cells. [77]. 

### 2.3. Macrophages

In many cancers, macrophages of the tumoral TME (Tumor Associated Macrophages: TAM) seems to support tumor progression. Steidl et. al. [78] found a macrophage gene signature correlated with the failure of primary treatment and then in an independent cohort of patients they demonstrated by immunohistochemistry (IHC), that higher number of CD68^+^ TAM was associated with shortened survival and with the outcome of secondary treatments such as autologous stem-cell transplantation [78]. Later many, but not all [79,80,81] IHC studies, conducted by Tan et. al., Greaves et. al., Tzankov A et. al., Gotti et. al., [82,83,84,85] confirmed the relationship among TAM and inferior outcomes after upfront treatment [82,83,84,85]. Moreover, the molecular characterization of HR cells reported in the neoplastic clone the over-expression of CSF1R (colony stimulating factor 1 receptor), a gene of the macrophage signature and the latter gene resulted associated with primary treatment failure [86]. Macrophages are versatile cells that can have an immune-stimulatory or an immune suppressive function [87]. Mantovani et. al. [87], to stigmatize the plasticity of macrophages, proposed their distinction in two functional sub-type: the M1 and the M2. The M1, or classically activated macrophages, includes mononuclear phagocytes activated by bacterial moieties and Th1 cytokines. The M2-type macrophages, or alternatively activated macrophages, express several proteins like CD206 (mannose receptor), CD204 (scavenger receptor A), IL-1, IL-10, CC ligand 22 (CCL22), and CD163 [87,88,89]. They suppress immune response, promote angiogenesis and tumor progression and metastasis. The M1 and M2 are present in the TME of cHL. Zaki et. al. [90] observed a correlation among number of M1 cells and favorable prognosis in mixed cellularity subtype of cHL [90].

In a recent study was described the cellular components of microenvironment underling topographical aspects of the immune-niche surround RS cells. According to this model, in TME was observed abundant PD-L1+TAMs and PD-1+ CD4 T cells, that were in contact with PD-L1+ tumor cells. These observations supported a possible role also of the macrophages in the mechanism of action of checkpoint inhibitor therapy [91].

### 2.4. Mast Cells

Inflammatory infiltrate of cHL contains also mast cells. These cells are positive for CD30L and tend to activate the RS cells through the interaction between CD30-CD30L [92,93]. Moreover, mast cells can promote tumor development thanks to the release of pro-angiogenic factor that increased the vasculature [94], the release of protease that enhance fibroblast proliferation and the secretion of tumor-promoting cytokines that induce an immunotolerant status of the TME [95]. 

The association between the number of mast cells and survival of patients with cHL is controversial. Molin et. al. [92] found a correlation between higher number of mast cells and poor prognosis [92], whereas retrospective study of 104 patients did not find any association between the degree of mast cells infiltration and outcome [96]. However, in further study Andersen et. al. [97] showed a significant association between high mast cells counts and poorer EFS as well as OS in mixed cellularity but not in nodular sclerosis histological subtype [97].

## 3. cHL and Immunoescape

RS cells have developed different mechanisms to escape from antitumoral immune response. Several studies reported the down regulation of molecules involved in antigen presentation to cytotoxic cells including HLA-I and HLA-II. The down regulation of HLA-I in Epstein-Barr virus (EBV)-cases or the presence of a polymorphism in HLA-I in EBV+ cases represent mechanisms that allow RS cells to escape from CD8-mediated cytotoxicity [98]. Furthermore, an association between the expression of HLA-G by RS cells, EBV negative status and absence of MHC-I was found. HLA-G is a ligand for inhibitory receptor presents on NK cells and other immune cells, may contribute to escape from immunosurveillance [99].

Moreover, alterations of gene CD58 contribute to immune evasion of RS cells. CD58 is relevant for the activation and adhesion of CTL and NK cells through the interaction between the protein and the receptor CD2 [100,101]. Numerous studies reported mutations of the CD58-gene in HL, with concurrent loss of expression of the protein [102,103,104,105]. CD58 mutations are observed in three Hodgkin’s lymphoma cell lines by whole-exome sequencing [104]. Probably the absence of CD58 allow RS cells evasion from immune recognition, particularly during advanced disease when the tumor cells become less dependent on the immune infiltrate and more immunogenic [102].

APC cells use the CD137 receptor/ligand system to costimulate activated T cells and the ectopic expression and release of CD137 by RS impaired T cells activation [106]. Many other molecules secreted by H/RS such as PGE2, LGALS1 and TGFβ are immunosuppressive factors [57,107,108]. The immunosuppressive activity is also mediated by the low levels of natural killer group 2D (NKG2D) expressed by RS [109]. The bond between NKG2DL and his receptor modulates lymphocyte activation and promotes immunity [109].

Another important mechanism by which RS cells evade host immuneresponse is by the expression of FASL, which induce apoptosis of activated CD8^+^ and Th1 cells [98]. RS also express FAS, but the tumor cells, thanks to mutations of the FAS gene, are resistant to the induction of apoptosis mediated by FAS-FASL complex. [110,111,112].

Moreover, in HL there is an overexpression of programmed cell death ligand 1 (PD-L1) and ligand 2 (PD-L2) [113,114]. RS cells present membranous expression of PD-L1 and the amplification of a region 9p24.1 that includes PD-L1 and PD-L2 explains the overexpression of PD-Ls in more than 85% of cHL patients [113]. For Roemer et al. PD-L1/PD-L2 alterations are a defining feature of cHL, 97% of the patients had concordant alterations of the PD-L1 and PD-L2 loci (including polysomy, copy gain and amplification) and this amplification was connected to a short PFS and a more advanced stage of disease [115]. In addition, alternative mechanisms promoting overexpression of PD-L1 and PD-L2 by RS include amplification of gene JAK2, leading to JAK2 protein overexpression and subsequent transcriptional activation of PD-L1, Epstein-Barr virus infection and activation of AP-1 [113,116]. Moreover Steidl et al. found a highly expressed chromosomal fusion gene involving CIITA, that caused the downregulation of MHCII expression and overexpression of PD-L1 and PD-L2 [117].

The binding of PD-1 ligands to PD-1, present on T cells, has been shown to contribute to the inhibition of the antitumoral function [114]. This phenomenon is called T cell “exhaustion” and is associated with an altered metabolism and singular transcriptional program compared with memory and effector T cells. A main characteristic of T cell exhaustion is the elevated expression of multiple inhibitory co-receptors, including PD-1, cytotoxic T lymphocyte antigen-4 (CTLA-4), lymphocyte-activation gene 3 (Lag-3) T cells immunoglobulin and mucin domain 3 (Tim-3) [118]. These group of proteins called “immune checkpoints”, in physiologic conditions limit aberrant immune cell activities following chronic activation or preventing autoimmune responses, but in the setting of cancer TME they diminish immune responses against malignant cells, allowing to the latter the escape from immune surveillance [119].

## 4. GEP Signature and TME

Previous immunohistochemistry (IHC) studies, have described the morphology and immunophenotype of TME. However, IHC results published generally are based on the use of single IHC biomarker that does not show simultaneously the predictive role of multiple cellular elements in the TME. Moreover, IHC technique presented interpretation bias and limitations.

**Figure 2:**
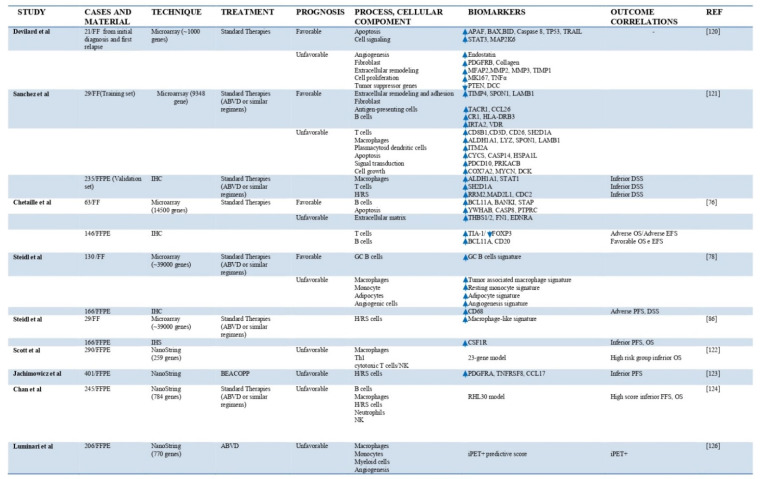
Published studies examining GEP and outcome in classical Hodgkin lymphoma.

Several groups have tried to define the molecular characteristics of the TME through high throughput technologies. Gene expression-profiling (GEP) results could be used to simultaneously measure the expression levels of thousands of gene. In many cases, the GEP results provided quantity genes level to building a gene expression signature suggesting how the cellular components of TME influenced the state of disease. Furthermore, expression array represented an opportunity to discover gene expression signatures associated with treatment outcomes (Figure 2).

A first group profiled the total tissue of 21 cases of cHL. In this study, they observed different transcriptional patterns with distinct response to therapy and clinical outcome. They noted how genes involved to fibroblast activation or function, but also to angiogenesis extracellular matrix remodeling and cell proliferation were overspread in sample with bad outcome. In the same group most genes related to tumor suppressor were underexpressed. While patient with a good prognosis were characterized by the up regulation of genes involved in apoptosis activation and cell signaling [120].

Sanchez et al. [121] identified specific gene signatures associated with outcome. Gene expressed by a group of T cells, macrophages and plasmacytoid dendritic cells were overexpressed in group of patients with unfavorable outcome. In the same unfavorable outcome groups, other genes related to apoptosis, signal transduction and cell growth were overspread. Using immunohistochemical analysis, representative markers of the immune response signatures and cell-cycle signatures were validated in an independent cohort. In agreement with the data of the gene expression, the results demonstrated a relation between increased number of macrophages markers (ALDH1, LYZ), T cells (SH2D1A) and inferior disease specific survival (DSS) [121].

Later, Chetaille et al. [76] identified new prognostic factors in cases of cHL with EBV infection. Gene profiling data showed in EBV+ cHL, Th1 activity and overexpression of macrophage genes. They also searched an association between outcome and specific gene expression signatures. In the group of patients with favorable outcome, they noted an overexpression of genes for B cells and genes involved in apoptotic pathway. To validate the signatures, they performed IHC analysis in an independent series of 146 cHL samples. High percentage of either TIA-1 reactive cells or topo-II positive cells had an adverse influence on OS. Whereas high count of BCL11A^+^, CD20^+^, FOXP3^+^ reactive cells showed a favorable influence [76].

Another study was designed to find a prognostic model from 130 frozen samples obtained from patients with cHL. Although, the results of unsupervisied hierarchical clustering analysis did not identify an association with the effect of treatment, the study of Steidl et al. provided a relation between a microenvironment gene signature and outcome. In the treatment-failure group, they observed an overexpression of gene signatures of TAMs, monocytes, adipocytes and for angiogenic cells. Using immunoistochemical analysis, they tried to confirm the findings of the gene expression analysis, the results showed an increased number of CD68^+^ cells associated with an adverse outcome [78]. Later, the same authors, investigated the gene expression profiles of microdissected H/RS cells and identified a link between the transcriptional program of the tumor cells and microenvironment. A signature of macrophage function in H/RS cells was correlated with first-line treatment failure. They focused their attention to CSF1R, a representative gene of macrophages signature. In an independent patient cohort, CSF1R *ISH* expressed by H/RS was correlated with inferior PFS, OS and the abundance of macrophages in microenvironment [86].

A new probe-based technology, the NanoString nCounter platform allow gene expression quantification using also low amounts of highly fragmented RNA isolated from routinely formalin-fixed paraffin embedded biopsies FFPE. NanoString method is based on direct measurement of gene expression level, eliminating enzymatic reactions and amplification bias. NanoString’s nCounter chemistry utilizes target-specific probes, collectively referred to as a CodeSet, that directly hybridize to a target of interest.

Scott et. al. [122] developed a predictive model of OS associated with outcomes in advanced stage cHL, the levels of gene expression were determined with NanoString Platform. 259 genes were selected from data of literature previously reported to be associated with outcome in cHL. Among these genes, a model with 23 genes was generated, involving components of the microenvironment and tumor. The study was conducted in 290 patients with advanced stage enrolled onto the E2496 intergroup trial company ABVD and Stanford regimes. The model and the threshold were tested in a validation cohort of patients with advanced stage cHL. Gene associated with macrophages program, activation of Th1 response, cytotoxic T cells/NK were overexpressed in patients with an increased risk of death [122].

In a recent study, the same group, applied the previously published 23-gene in a distinct cohort of 401 patients with advanced-stage cHL, treated with BEACOPP based regimens. The 23-gene predictor was not prognostic for PFS and OS in the context of BEACOPP-treated advanced stage cHL.

However, they identified that three individual genes PDGFRA, TNFRSF8 and CCL17 after multiple testing, were correlated with PFS in patients treated with BEACOPP based regimens.

This result highlighted how different therapeutic approaches may require the necessity to develop different predictors for risk assessment [123].

Another gene expression analysis explored the TME composition of 245 FFPE samples with cHL, including 71 paired primary and relapse specimens, to investigate temporal gene expression difference and association with post autologous stem cell transplant (ASCT) outcomes. Chan et al. observed a TME dynamism between primary and relapse specimens, moreover they showed that the biology at relapse, compared with primary diagnosis, contained more prognostic information for predicting treatment outcomes after ASCT. The authors developed a new prognostic model, RHL30 based on the expression of gene associated with tumor cells and immune cells type of TME (macrophage, neutrophil and natural killer). A high RHL30 score identified patients with unfavorable outcomes (worse FFS and OS) after ASCT [124].

Later, the same authors validated the RHL30 assay, in an additional independent cohort of 41 patients with relapsed cHL. In part, the latest results were different from those presented in the first work. The RHL30 risk score was associated with FFS post-ASCT, but the same cohort of patients didn’t present an association with OS [125].

The Interim PET (iPET), after 2 cycles of Chemotherapy is a good predictor of outcome in cHL.

Luminari et. al. [126], identified the biological features of patients iPET+ developing 13-gene signature. They evaluated the expression profile by NanoString using a commercial panel of 770 genes, filtered the 241 genes differentially expressed and developed a stringent gene signature. The authors found a predictive score associated with iPET status composed of genes (ITGA5, SAA1, CXCL2, SPP1, and TREM1) and Lymphocytes T-monocytes ratio (LMR) with the aim to define the right treatment strategy upfront without waiting two months from treatment start [126].

In a retrospective study was studied the association of 25-hidroxy vitamin D (VitD) blood level with data of gene expression also in cHL. VitD deficiency reactivated genes that mediate tumor cell survival and resistance to stress, contributing to promote cHL aggressiveness [127].

## 5. New TME Based Therapeutic Strategy

Approximately 80% of patients are cured with standard first line chemotherapy [128]. In patients with early-stage, the first line therapy made up of cycles of Adriamycin, Bleomycin, Vinblastine Sulfate, Dacarbazine (ABVD) chemotherapy, followed by radiotherapy in some cases. While Patients with advanced-stage disease usually receive a prolonged or more intense chemotherapy consisting of either ABVD or a regimen of bleomycin, etoposide, doxorubicin, cyclophosphamide, vincristine, procarbazine, and prednisone (BEACOPP), with the possible inclusion of radiation treatment [129].

However 15% of patients with early stage disease and 30% with advanced stage disease relapse or have primary refractory disease after initial treatment [128,130].

Patients with relapsed or refractory are treated with salvage chemotherapy followed by ASCT [131].

Brentuximab-vedotin (BV) a monoclonal antibody directed against the CD30 expressed by HRS cells, is another therapeutic opportunity for the treatment of cHL [132]. Originally BV has been used as second line therapy or a consolidation of the ASCT in high risk patient [133] recently BV was proposed into frontline treatment [134].

In this scenario, the growing interest in tumor TME has contributed to the development of exciting novel therapy that target not only the neoplastic cells but also the cellular components of the background. There are antibodies direct against PD-1 or PD-L1 and CTLA-4, which reverse the down regulation of T cell function induced by tumor condition and allow T cells activation in the TME.

Nivolumab is a fully immunoglobulin IgG4 anti-PD-1antibody and blocks the binding between PD-1 and PD-L1/PD-L2. Nivolumab has been studied in 23 patients with Hodgkin’s lymphoma, 78% were enrolled in the study after a relapse following autologous stem-cell transplantation and 78% after a relapse following the receipt of BV (CheckMate-039). The monoclonal antibody had an ORR of 87%, a CR rate of 17%, a partial response (PR) of 70% and PFS of 86% at 24 weeks [135]. Given these encouraging preliminary results, a subsequent phase II study (CheckMate-205) was conducted on 80 patients, who had received prior ASCT and BV. Nivolumab was administered at the dose of 3 mg/kg every 2 weeks and demonstrated a therapeutic activity with ORR of 66% at a median follow-up of 8,9 months [136]. Based on these results, FDA approved nivolumab for the treatment of patients with cHL for whom ASCT and BV failed.

Pembrolizumab is another IgG4 monoclonal antibody to PD-1. In a phase 1b multicenter study (KEYNOTE-013), pembrolizumab was administered at the dose of 10 mg/kg every 2 weeks for up to 2 years, to 31 patients with cHL unresponsive to treatment with BV (100%) and with relapsed after ASCT (71%). The ORR was of 65%, the CR rate was of 16% and PR rate was of 48%. The PFS was 69% at 24 weeks and 46% at 52 weeks. The treatment with pembrolizumab was well tolerated [137].

A study in phase II (KEYNOTE-087) was designed to evaluate the clinical activity of pembrolizumab in 3 cohorts of patients with relapsed/refractory cHL. In all cohorts were observed substantial clinical activity and a high ORR. Pembrolizumab received FDA approval for the treatment of adult and pediatric patients with refractory cHL, or those who have relapsed after three or more prior lines of therapy [138,139].

Results of long term follow up analysis of KEYNOTE-013 [140] nd KEYNOTE-087 [141] showed durable antitumor anticity of Pembrolizumab in phase 1 and phase 2 studies.

In solid tumors, antibodies anti-CTLA-4 were the first immune checkpoint inhibitors to receive FDA approval for the treatment of melanoma. In addition, also antibodies against PD-1 have been approved for the treatment of melanoma and non-small-cell-lung cancer [142]. CTLA-4 is a negative regulator of T cell activation and a CD28 homologue [143]. CTLA-4 interacts with CD80 and CD86 with a higher affinity than CD28, leading to impaired T cell costimulation and functional inactivation [144]. CTLA-4 is expressed by T cells, B cells, fibroblasts, but is unclear the role of CTLA-4 for non-T cells [145]. Ipilimumab is an antibody anti-CTLA-4 and it was the first checkpoint inhibitor examined in patients with HL. In a phase I study of 29 patients with hematologic malignancies, that also including 14 patients with relapsed/refractory HL after allogenic stem cell transplantation were treated with anti-CTLA-4. Ipilimumab was well tolerated, case of relevant graft-versus-host disease was absent and 2 patients showed a complete remission (CR) [146]. CTLA4 antibodies have been surpassed by antibodies direct against PD-1 and PD-L1, but they have been affective in combination therapies with other agents [147].

Other innovative therapeutic strategies have been developed targeting TAM and NK [148]. In a phase 1/2 trial was evaluated the effect of JNJ-40346527, an inhibitor of CSF1R expressed by RS cells and correlated to an increased number of TAM [86,149]. CSF1R inhibitor presented limited activity when used in the relapsed/refractory setting [150].

AFM13 is a bispecific antibodies against CD30 and CD16A, that recruits NK cells via binding to CD16A [151]. A phase I study (AFM13-101) was conducted in patients with cHL who relapsed or were refractory after standard therapies [152].

Although the AFM13 represents a new target therapy, the study demonstrated limited efficacy (ORR 12% to 23%, no CR) [152].

Even in patients with relapsed cHL, chimeric antigen receptor T cells (CAR T) represent a potential treatment. CAR T therapy against CD30 has not produced encouraging results [153]. While anti-CD123 CAR T cells for the treatment of cHL represents a more promising CAR T cell approach [154]. CD123 is expressed on RS cells, macrophages, plasmacytoid dendritic cells, eosinophils, basophils, and mast cells [155]. In cHL CD123 CAR T were capable of targeting malignant cells and TAMs eliminating a crucial immunosuppressive component of TME [154].

## References

[1] Swerdlow S.H., Campo E., Harris N.L., Jaffe E.S., Pileri S.A., Stein H., Thiele J. (2017). WHO Classification of Tumours of Haematopoietic and Lymphoid Tissues.

[2] Newcom S.R., Kadin M.E., Phillips C. (1988). L-428 Reed-Sternberg cells and mononuclear Hodgkin’s cells arise from a single cloned mononuclear cell. Int. J. Cell Cloning.

[3] Rengstl B., Newrzela S., Heinrich T., Weiser C., Thalheimer F.B., Schmid F., Warner K., Hartmann S., Schroeder T., Küppers R. (2013). Incomplete cytokinesis and re-fusion of small mononucleated Hodgkin cells lead to giant multinucleated Reed-Sternberg cells. Proc. Natl. Acad. Sci. USA.

[4] Küppers R., Schwering I., Bräuninger A., Rajewsky K., Hansmann M.L. (2002). Biology of Hodgkin’s lymphoma. Ann. Oncol..

[5] Jaffe E.S., Harris N.L., Stein H., Vardiman J. (2001). Tumours of Haematopoietic and Lymphoid Tissues.

[6] Swerdlow S.H., Campo E., Harris N.L., Jaffe E.S., Pileri S.A., Stein H., Thiele J., Vardiman J. (2008). WHO Classification of Tumours of Haematopoietic and Lymphoid Tissues.

[7] Schmitz R., Stanelle J., Hansmann M.-L., Küppers R. (2009). Pathogenesis of Classical and Lymphocyte-Predominant Hodgkin Lymphoma. Annu. Rev. Pathol. Mech. Dis..

[8] Ushmorov A., Leithäuser F., Sakk O., Weinhaüsel A., Popov S.W., Möller P., Wirth T. (2006). Epigenetic processes play a major role in B-cell-specific gene silencing in classical Hodgkin lymphoma. Blood.

[9] Thévenin C., Lucas B.P., Kozlow E.J., Kehrl J.H. (1993). Cell type- and stage-specific expression of the CD20/B1 antigen correlates with the activity of a diverged octamer DNA motif present in its promote. J. Biol. Chem..

[10] Young R.L., Korsmeyer S.J. (1993). A Negative Regulatory Element in the bcl-2 5′-Untranslated Region Inhibits Expression from an Upstream Promoter. Mol. Cell. Biol..

[11] Kozmik Z., Wang S., Dörfler P., Adams B., Busslinger M. (1992). The promoter of the CD19 gene is a target for the B-cell-specific transcription factor BSAP. Mol. Cell. Biol..

[12] DeKoter R.P., Singh H. (2000). Regulation of B lymphocyte and macrophage development by graded expression of PU.1. Science.

[13] Stein H., Marafioti T., Foss H.D., Laumen H., Hummel M., Anagnostopoulos I., Wirth T., Demel G., Falini B. (2001). Down-regulation of BOB.1/OBF.1 and Oct2 in classical Hodgkin disease but not in lymphocyte predominant Hodgkin disease correlates with immunoglobulin transcription. Blood.

[14] Mathas S., Janz M., Hummel F., Hummel M., Wollert-Wulf B., Lusatis S., Anagnostopoulos I., Lietz A., Sigvardsson M., Jundt F. (2006). Intrinsic inhibition of transcription factor E2A by HLH proteins ABF-1 and Id2 mediates reprogramming of neoplastic B cells in Hodgkin lymphoma. Nat. Immunol..

[15] Jundt F., Anagnostopoulos I., Förster R., Mathas S., Stein H., Dörken B. (2002). Activated Notch1 signaling promotes tumor cell proliferation and survival in Hodgkin and anaplastic large cell lymphoma. Blood.

[16] Foss H.-D., Reusch R., Demel G., Lenz G., Anagnostopoulos I., Hummel M., Stein H. (1999). Frequent Expression of the B-Cell–Specific Activator Protein in Reed-Sternberg Cells of Classical Hodgkin’s Disease Provides Further Evidence for Its B-Cell Origin. Blood.

[17] Weniger M.A., Küppers R. (2016). NF-κB deregulation in Hodgkin lymphoma. Semin. Cancer Biol..

[18] Aldinucci D., Lorenzon D., Cattaruzza L., Pinto A., Gloghini A., Carbone A., Colombatti A. (2008). Expression of CCR5 receptors on Reed-Sternberg cells and Hodgkin lymphoma cell lines: Involvement of CCL5/Rantes in tumor cell growth and microenvironmental interactions. Int. J. Cancer.

[19] Chiu A., Xu W., He B., Dillon S.R., Gross J.A., Sievers E., Qiao X., Santini P., Hyjek E., Lee J.W. (2007). Hodgkin lymphoma cells express TACI and BCMA receptors and generate survival and proliferation signals in response to BAFF and APRIL. Blood.

[20] Fiumara P., Snell V., Li Y., Mukhopadhyay A., Younes M., Gillenwater A.M., Cabanillas F., Aggarwal B.B., Younes A. (2001). Functional expression of receptor activator of nuclear factor kappaB in Hodgkin disease cell lines. Blood.

[21] Schwab U., Stein H., Gerdes J., Lemke H., Kirchner H., Schaadt M., Diehl V. (1982). Production of a monoclonal antibody specific for Hodgkin and Sternberg-Reed cells of Hodgkin’s disease and a subset of normal lymphoid cells. Nature.

[22] Cabannes E., Khan G., Aillet F., Jarrett R.F., Hay R.T. (1999). Mutations in the IkBa gene in Hodgkin’s disease suggest a tumour suppressor role for IκBα. Oncogene.

[23] Emmerich F., Theurich S., Hummel M., Haeffker A., Vry M.S., Döhner K., Bommert K., Stein H., Dörken B. (2003). Inactivating I kappa B epsilon mutations in Hodgkin/Reed-Sternberg cells. J. Pathol..

[24] Jungnickel B., Staratschek-Jox A., Bräuninger A., Spieker T., Wolf J., Diehl V., Hansmann M.L., Rajewsky K., Küppers R. (2000). Clonal deleterious mutations in the iκbα gene in the malignant cells in Hodgkin’s lymphoma. J. Exp. Med..

[25] Otto C., Giefing M., Massow A., Vater I., Gesk S., Schlesner M., Richter J., Klapper W., Hansmann M.-L., Siebert R. (2012). Genetic lesions of the TRAF3 and MAP3K14 genes in classical Hodgkin lymphoma. Br. J. Haematol..

[26] Martín-Subero J.I., Gesk S., Harder L., Sonoki T., Tucker P.W., Schlegelberger B., Grote W., Novo F.J., Calasanz M.J., Hansmann M.L. (2002). Recurrent involvement of the REL and BCL11A loci in classical Hodgkin lymphoma. Blood.

[27] Scheeren F.A., Diehl S.A., Smit L.A., Beaumont T., Naspetti M., Bende R.J., Blom B., Karube K., Ohshima K., van Noesel C.J.M. (2008). IL-21 is expressed in Hodgkin lymphoma and activates STAT5: Evidence that activated STAT5 is required for Hodgkin lymphomagenesis. Blood.

[28] Baus D., Pfitzner E. (2006). Specific function of STAT3, SOCS1, and SOCS3 in the regulation of proliferation and survival of classical Hodgkin lymphoma cells. Int. J. Cancer.

[29] Kube D., Holtick U., Vockerodt M., Ahmadi T., Haier B., Behrmann I., Heinrich P.C., Diehl V., Tesch H. (2001). STAT3 is constitutively activated in Hodgkin cell lines. Blood.

[30] Skinnider B.F., Elia A.J., Gascoyne R.D., Patterson B., Trumper L., Kapp U., Mak T.W. (2002). Signal transducer and activator of transcription 6 is frequently activated in Hodgkin and Reed-Sternberg cells of Hodgkin lymphoma. Blood.

[31] Dutton A., Reynolds G.M., Dawson C.W., Young L.S., Murray P.G. (2005). Constitutive activation of phosphatidyl-inositide 3 kinase contributes to the survival of Hodgkin’s lymphoma cells through a mechanism involving Akt kinase and mTOR. J. Pathol..

[32] Georgakis G.V., Li Y., Rassidakis G.Z., Medeiros L.J., Mills G.B., Younes A. (2005). Inhibition of the phosphatidylinositol-3 kinase/Akt promotes G1 cell cycle arrest and apoptosis in Hodgkin lymphoma. Br. J. Haematol..

[33] Renne C., Willenbrock K., Küppers R., Hansmann M.-L., Bräuninger A. (2005). Autocrine- and paracrine-activated receptor tyrosine kinases in classic Hodgkin lymphoma. Blood.

[34] Zheng B., Fiumara P., Li Y.V., Georgakis G., Snell V., Younes M., Vauthey J.N., Carbone A., Younes A. (2003). MEK/ERK pathway is aberrantly active in Hodgkin disease: A signaling pathway shared by CD30, CD40, and RANK that regulates cell proliferation and survival. Blood.

[35] Aldinucci D., Celegato M., Casagrande N. (2016). Microenvironmental interactions in classical Hodgkin lymphoma and their role in promoting tumor growth, immune escape and drug resistance. Cancer Lett..

[36] Küppers R. (2009). The biology of Hodgkin’s lymphoma. Nat. Rev. Cancer.

[37] Aldinucci D., Gloghini A., Pinto A., De Filippi R., Carbone A. (2010). The classical Hodgkin’s lymphoma microenvironment and its role in promoting tumour growth and immune escape. J. Pathol..

[38] Samoszuk M., Nansen L. (1990). Detection of interleukin-5 messenger RNA in Reed-Sternberg cells of Hodgkin’s disease with eosinophilia. Blood.

[39] Hanamoto H., Nakayama T., Miyazato H., Takegawa S., Hieshima K., Tatsumi Y., Kanamaru A., Yoshie O. (2004). Expression of CCL28 by Reed-Sternberg cells defines a major subtype of classical Hodgkin’s disease with frequent infiltration of eosinophils and/or plasma cells. Am. J. Pathol..

[40] Cattaruzza L., Gloghini A., Olivo K., Di Francia R., Lorenzon D., De Filippi R., Carbone A., Colombatti A., Pinto A., Aldinucci D. (2009). Functional coexpression of Interleukin (IL)-7 and its receptor (IL-7R) on Hodgkin and Reed-Sternberg cells: Involvement of IL-7 in tumor cell growth and microenvironmental interactions of Hodgkin’s lymphoma. Int. J. Cancer.

[41] Fischer M., Juremalm M., Olsson N., Backlin C., Sundström C., Nilsson K., Enblad G., Nilsson G. (2003). Expression of CCL5/RANTES by Hodgkin and reed-sternberg cells and its possible role in the recruitment of mast cells into lymphomatous tissue. Int. J. Cancer.

[42] Baumforth K.R.N., Birgersdotter A., Reynolds G.M., Wei W., Kapatai G., Flavell J.R., Kalk E., Piper K., Lee S., Machado L. (2008). Expression of the Epstein-Barr virus-encoded Epstein-Barr virus nuclear antigen 1 in Hodgkin’s lymphoma cells mediates Up-regulation of CCL20 and the migration of regulatory T cells. Am. J. Pathol..

[43] Küppers R. (2005). Mechanisms of B-cell lymphoma pathogenesis. Nat. Rev. Cancer.

[44] Aldinucci D., Olivo K., Lorenzon D., Poletto D., Gloghini A., Carbone A., Pinto A. (2005). The role of interleukin-3 in classical Hodgkin’s disease. Leuk. Lymphoma.

[45] Moore K.W., de Waal Malefyt R., Coffman R.L., O’Garra A. (2001). Interleukin-10 and the interleukin-10 receptor. Annu. Rev. Immunol..

[46] Theill L.E., Boyle W.J., Penninger J.M. (2002). RANK-L and RANK: T cells, bone loss, and mammalian evolution. Annu. Rev. Immunol..

[47] Hsu S.M., Hsu P.L. (1994). The nature of Reed-Sternberg cells: Phenotype, genotype, and other properties. Crit. Rev. Oncog..

[48] Stein R., Qu Z., Cardillo T.M., Chen S., Rosario A., Horak I.D., Hansen H.J., Goldenberg D.M. (2004). Antiproliferative activity of a humanized anti-CD74 monoclonal antibody, hLL1, on B-cell malignancies. Blood.

[49] Foss H.D., Herbst H., Gottstein S., Demel G., Araujó I., Stein H. (1996). Interleukin-8 in Hodgkin’s disease. Preferential expression by reactive cells and association with neutrophil density. Am. J. Pathol..

[50] Luciani M.G., Stoppacciaro A., Peri G., Mantovani A., Ruco L.P. (1998). The monocyte chemotactic protein a (MCP-1) and interleukin 8 (IL-8) in Hodgkin’s disease and in solid tumours. Mol. Pathol..

[51] Enblad G., Molin D., Glimelius I., Fischer M., Nilsson G. (2007). The Potential Role of Innate Immunity in the Pathogenesis of Hodgkin’s Lymphoma. Hematol. Oncol. Clin. N. Am..

[52] Wein F., Küppers R. (2015). The role of T cells in the microenvironment of Hodgkin lymphoma. J. Leukoc. Biol..

[53] Liu Y., Sattarzadeh A., Diepstra A., Visser L., van den Berg A. (2014). The microenvironment in classical Hodgkin lymphoma: An actively shaped and essential tumor component. Semin. Cancer Biol..

[54] Aldinucci D., Gloghini A., Pinto A., Colombatti A., Carbone A. (2012). The role of CD40/CD40L and interferon regulatory factor 4 in Hodgkin lymphoma microenvironment. Leuk. Lymphoma.

[55] Veldman J., Visser L., Huberts-Kregel M., Muller N., Hepkema B., van den Berg A., Diepstra A. (2020). Rosetting T cells in Hodgkin lymphoma are activated by immunological synapse components HLA class II and CD58. Blood.

[56] Poppema S., Potters M., Emmens R., Visser L., van den Berg A. (1999). Immune reactions in classical Hodgkin’s lymphoma. Semin. Hematol..

[57] Skinnider B.F., Mak T.W. (2002). The Role of Cytokines in Classical Hodgkin Lymphoma. Blood.

[58] Balkwill F., Mantovani A. (2001). Inflammation and cancer: Back to Virchow?. Lancet.

[59] Greaves P., Clear A., Owen A., Iqbal S., Lee A., Matthews J., Wilson A., Calaminici M., Gribben J.G. (2013). Defining characteristics of classical Hodgkin lymphoma microenvironment T-helper cells. Blood.

[60] Álvaro T., Lejeune M., Salvadó M.T., Bosch R., García J.F., Jaén J., Banham A.H., Roncador G., Montalbán C., Piris M.A. (2005). Outcome in Hodgkin’s lymphoma can be predicted from the presence of accompanying cytotoxic and regulatory T cells. Clin. Cancer Res..

[61] Poppema S., Bhan A.K., Reinherz E.L., Posner M.R., Schlossman S.F. (1982). In situ immunologic characterization of cellular constituents in lymph nodes and spleens involved by Hodgkin’s disease. Blood.

[62] Alvaro-Naranjo T., Lejeune M., Salvadó-Usach M.T., Bosch-Príncep R., Reverter-Branchat G., Jaén-Martínez J., Pons-Ferré L.E. (2005). Tumor-infiltrating cells as a prognostic factor in Hodgkin’s lymphoma: A quantitative tissue microarray study in a large retrospective cohort of 267 patients. Leuk. Lymphoma.

[63] Kelley T.W., Pohlman B., Elson P., Hsi E.D. (2007). The ratio of FOXP3+ regulatory T cells to granzyme B+ cytotoxic T/NK cells predicts prognosis in classical Hodgkin lymphoma and is independent of bcl-2 and MAL expression. Am. J. Clin. Pathol..

[64] Hori S., Nomura T., Sakaguchi S. (2003). Control of Regulatory T Cell Development by the Transcription Factor Foxp3. Science.

[65] Cretney E., Kallies A., Nutt S.L. (2013). Differentiation and function of Foxp3+ effector regulatory T cells. Trends Immunol..

[66] Shevach E.M. (2009). Mechanisms of Foxp3+ T Regulatory Cell-Mediated Suppression. Immunity.

[67] Tang Q., Bluestone J.A. (2008). The Foxp3+ regulatory T cell: A jack of all trades, master of regulation. Nat. Immunol..

[68] Vignali D.A.A., Collison L.W., Workman C.J. (2008). How regulatory T cells work. Nat. Rev. Immunol..

[69] Bates G.J., Fox S.B., Han C., Leek R.D., Garcia J.F., Harris A.L., Banham A.H. (2006). Quantification of regulatory T cells enables the identification of high-risk breast cancer patients and those at risk of late relapse. J. Clin. Oncol..

[70] Curiel T.J., Coukos G., Zou L., Alvarez X., Cheng P., Mottram P., Evdemon-Hogan M., Conejo-Garcia J.R., Zhang L., Burow M. (2004). Specific recruitment of regulatory T cells in ovarian carcinoma fosters immune privilege and predicts reduced survival. Nat. Med..

[71] Gao Q., Qiu S.-J., Fan J., Zhou J., Wang X.-Y., Xiao Y.-S., Xu Y., Li Y.-W., Tang Z.-Y. (2007). Intratumoral balance of regulatory and cytotoxic T cells is associated with prognosis of hepatocellular carcinoma after resection. J. Clin. Oncol..

[72] Petersen R.P., Campa M.J., Sperlazza J., Conlon D., Joshi M.-B., Harpole D.H., Patz E.F. (2006). Tumor infiltrating Foxp3+ regulatory T-cells are associated with recurrence in pathologic stage I NSCLC patients. Cancer.

[73] Perrone G., Ruffini P.A., Catalano V., Spino C., Santini D., Muretto P., Spoto C., Zingaretti C., Sisti V., Alessandroni P. (2008). Intratumoural FOXP3-positive regulatory T cells are associated with adverse prognosis in radically resected gastric cancer. Eur. J. Cancer.

[74] Shah W., Yan X., Jing L., Zhou Y., Chen H., Wang Y. (2011). A reversed CD4/CD8 ratio of tumor-infiltrating lymphocytes and a high percentage of CD4^+^FOXP3^+^ regulatory T cells are significantly associated with clinical outcome in squamous cell carcinoma of the cervix. Cell. Mol. Immunol..

[75] Vardhana S., Younes A. (2016). The immune microenvironment in hodgkin lymphoma: T cells, B cells, and immune checkpoints. Haematologica.

[76] Chetaille B., Bertucci F., Finetti P., Esterni B., Stamatoullas A., Picquenot J.M., Copin M.C., Morschhauser F., Casasnovas O., Petrella T. (2009). Molecular profiling of classical Hodgkin lymphoma tissues uncovers variations in the tumor microenvironment and correlations with EBV infection and outcome. Blood.

[77] Inoue S., Leitner W.W., Golding B., Scott D. (2006). Inhibitory effects of B cells on antitumor immunity. Cancer Res..

[78] Steidl C., Lee T., Shah S.P., Farinha P., Han G., Nayar T., Delaney A., Jones S.J., Iqbal J., Weisenburger D.D. (2010). Tumor-Associated Macrophages and Survival in Classic Hodgkin’s Lymphoma. N. Engl. J. Med..

[79] Azambuja D., Natkunam Y., Biasoli I., Lossos I.S., Anderson M.W., Morais J.C., Spector N. (2012). Lack of association of tumor-associated macrophages with clinical outcome in patients with classical Hodgkin’s lymphoma. Ann. Oncol..

[80] Harris J.A., Jain S., Ren Q., Zarineh A., Liu C., Ibrahim S. (2012). CD163 versus CD68 in tumor associated macrophages of classical hodgkin lymphoma. Diagn. Pathol..

[81] Kayal S., Mathur S., Karak A.K., Kumar L., Sharma A., Bakhshi S., Raina V. (2014). CD68 tumor-associated macrophage marker is not prognostic of clinical outcome in classical Hodgkin lymphoma. Leuk. Lymphoma.

[82] Tan K.L., Scott D.W., Hong F., Kahl B.S., Fisher R.I., Bartlett N.L., Advani R.H., Buckstein R., Rimsza L.M., Connors J.M. (2012). Tumor-associated macrophages predict inferior outcomes in classic Hodgkin lymphoma: A correlative study from the E2496 Intergroup trial. Blood.

[83] Greaves P., Clear A., Coutinho R., Wilson A., Matthews J., Owen A., Shanyinde M., Lister T.A., Calaminici M., Gribben J.G. (2013). Expression of FOXP3, CD68, and CD20 at Diagnosis in the Microenvironment of Classical Hodgkin Lymphoma Is Predictive of Outcome. J. Clin. Oncol..

[84] Tzankov A., Matter M.S., Dirnhofer S. (2010). Refined Prognostic Role of CD68-Positive Tumor Macrophages in the Context of the Cellular Micromilieu of Classical Hodgkin Lymphoma. Pathobiology.

[85] Gotti M., Nicola M., Lucioni M., Fiaccadori V., Ferretti V., Sciarra R., Costanza M., Bono E., Molo S., Maffi A. (2016). Independent prognostic impact of tumour-infiltrating macrophages in early-stage Hodgkin’s lymphoma. Hematol. Oncol..

[86] Steidl C., Diepstra A., Lee T., Chan F.C., Farinha P., Tan K., Telenius A., Barclay L., Shah S.P., Connors J.M. (2012). Gene expression profiling of microdissected Hodgkin Reed-Sternberg cells correlates with treatment outcome in classical Hodgkin lymphoma. Blood.

[87] Allavena P., Mantovani A. (2012). Immunology in the clinic review series; focus on cancer: Tumour-associated macrophages: Undisputed stars of the inflammatory tumour microenvironment. Clin. Exp. Immunol..

[88] Jensen T.O., Schmidt H., Møller H.J., Høyer M., Maniecki M.B., Sjoegren P., Christensen I.J., Steiniche T. (2009). Macrophage markers in serum and tumor have prognostic impact in American Joint Committee on Cancer stage I/II melanoma. J. Clin. Oncol..

[89] Ma J., Liu L., Che G., Yu N., Dai F., You Z. (2010). The M1 form of tumor-associated macrophages in non-small cell lung cancer is positively associated with survival time. BMC Cancer.

[90] Zaki M.A.A., Wada N., Ikeda J., Shibayama H., Hashimoto K., Yamagami T., Tatsumi Y., Tsukaguchi M., Take H., Tsudo M. (2011). Prognostic implication of types of tumor-associated macrophages in Hodgkin lymphoma. Virchows Arch..

[91] Carey C.D., Gusenleitner D., Lipschitz M., Roemer M.G.M., Stack E.C., Gjini E., Hu X., Redd R., Freeman G.J., Neuberg D. (2017). Topological analysis reveals a PD-L1-associated microenvironmental niche for Reed-Sternberg cells in Hodgkin lymphoma. Blood.

[92] Molin D., Edstrom A., Glimelius I., Glimelius B., Nilsson G., Sundstrom C., Enblad G. (2002). Mast cell infiltration correlates with poor prognosis in Hodgkin’s lymphoma. Br. J. Haematol..

[93] Molin D., Fischer M., Xiang Z., Larsson U., Harvima I., Venge P., Nilsson K., Sundström C., Enblad G., Nilsson G. (2001). Mast cells express functional CD30 ligand and are the predominant CD30L-positive cells in Hodgkin’s disease. Br. J. Haematol..

[94] Mizuno H., Nakayama T., Miyata Y., Saito S., Nishiwaki S., Nakao N., Takeshita K., Naoe T. (2012). Mast cells promote the growth of Hodgkin’s lymphoma cell tumor by modifying the tumor microenvironment that can be perturbed by bortezomib. Leukemia.

[95] Oldford S.A., Marshall J.S. (2015). Mast cells as targets for immunotherapy of solid tumors. Mol. Immunol..

[96] Keresztes K., Szollosi Z., Simon Z., Tarkanyi I., Nemes Z., Illes A. (2007). Retrospective Analysis of the Prognostic Role of Tissue Eosinophil and Mast Cells in Hodgkin’s Lymphoma. Pathol. Oncol. Res..

[97] Andersen M.D., Kamper P., Nielsen P.S., Bendix K., Riber-Hansen R., Steiniche T., Hamilton-Dutoit S., Clausen M., d’Amore F. (2016). Tumour-associated mast cells in classical Hodgkin’s lymphoma: Correlation with histological subtype, other tumour-infiltrating inflammatory cell subsets and outcome. Eur. J. Haematol..

[98] Poppema S. (2005). Immunobiology and pathophysiology of Hodgkin lymphomas. Hematol. Am. Soc. Hematol. Educ. Progr..

[99] Diepstra A., Poppema S., Boot M., Visser L., Nolte I.M., Niens M., Te Meerman G.J., Van Den Berg A. (2008). HLA-G protein expression as a potential immune escape mechanism in classical Hodgkin’s lymphoma. Tissue Antigens.

[100] Altomonte M., Gloghini A., Bertola G., Gasparollo A., Carbone A., Ferrone S., Maio M. (1993). Differential expression of cell adhesion molecules CD54/CD11a and CD58/CD2 by human melanoma cells and functional role in their interaction with cytotoxic cells. Cancer Res..

[101] Gwin J.L., Gercel-Taylor C., Taylor D.D., Eisenberg B. (1996). Role of LFA-3, ICAM-1, and MHC Class I on the Sensitivity of Human Tumor Cells to LAK Cells. J. Surg. Res..

[102] Schneider M., Schneider S., Zühlke-Jenisch R., Klapper W., Sundström C., Hartmann S., Hansmann M.-L., Siebert R., Küppers R., Giefing M. (2015). Alterations of the CD58 gene in classical Hodgkin lymphoma. Genes. Chromosomes Cancer.

[103] Reichel J., Chadburn A., Rubinstein P.G., Giulino-Roth L., Tam W., Liu Y., Gaiolla R., Eng K., Brody J., Inghirami G. (2015). Flow sorting and exome sequencing reveal the oncogenome of primary Hodgkin and Reed-Sternberg cells. Blood.

[104] Liu Y., Abdul Razak F.R., Terpstra M., Chan F.C., Saber A., Nijland M., van Imhoff G., Visser L., Gascoyne R., Steidl C. (2014). The mutational landscape of Hodgkin lymphoma cell lines determined by whole-exome sequencing. Leukemia.

[105] Abdul Razak F.R., Diepstra A., Visser L., van den Berg A. (2016). CD58 mutations are common in Hodgkin lymphoma cell lines and loss of CD58 expression in tumor cells occurs in Hodgkin lymphoma patients who relapse. Genes Immun..

[106] Ho W.T., Pang W.L., Chong S.M., Castella A., Al-Salam S., Tan T.E., Moh M.C., Koh L.K., Gan S.U., Cheng C.K. (2013). Expression of CD137 on Hodgkin and Reed-Sternberg cells inhibits T-cell activation by eliminating CD137 ligand expression. Cancer Res..

[107] Chemnitz J.M., Driesen J., Classen S., Riley J.L., Debey S., Beyer M., Popov A., Zander T., Schultze J.L. (2006). Prostaglandin E2 Impairs CD4^+^ T Cell Activation by Inhibition of lck: Implications in Hodgkin’s Lymphoma. Cancer Res..

[108] Juszczynski P., Ouyang J., Monti S., Rodig S.J., Takeyama K., Abramson J., Chen W., Kutok J.L., Rabinovich G.A., Shipp M.A. (2007). The AP1-dependent secretion of galectin-1 by Reed Sternberg cells fosters immune privilege in classical Hodgkin lymphoma. Proc. Natl. Acad. Sci. USA.

[109] Spear P., Wu M.-R., Sentman M.-L., Sentman C.L. (2013). NKG2D ligands as therapeutic targets. Cancer Immun..

[110] Maggio E.M., Van Den Berg A., de Jong D., Diepstra A., Poppema S. (2003). Low frequency of FAS mutations in Reed-Sternberg cells of Hodgkin’s lymphoma. Am. J. Pathol..

[111] Thomas R.K., Kallenborn A., Wickenhauser C., Schultze J.L., Draube A., Vockerodt M., Re D., Diehl V., Wolf J. (2002). Constitutive Expression of c-FLIP in Hodgkin and Reed-Sternberg Cells. Am. J. Pathol..

[112] Mathas S., Lietz A., Anagnostopoulos I., Hummel F., Wiesner B., Janz M., Jundt F., Hirsch B., Jöhrens-Leder K., Vornlocher H.-P. (2004). c-FLIP mediates resistance of Hodgkin/Reed-Sternberg cells to death receptor-induced apoptosis. J. Exp. Med..

[113] Green M.R., Monti S., Rodig S.J., Juszczynski P., Currie T., O’Donnell E., Chapuy B., Takeyama K., Neuberg D., Golub T.R. (2010). Integrative analysis reveals selective 9p24.1 amplification, increased PD-1 ligand expression, and further induction via JAK2 in nodular sclerosing Hodgkin lymphoma and primary mediastinal large B-cell lymphoma. Blood.

[114] Yamamoto R., Nishikori M., Kitawaki T., Sakai T., Hishizawa M., Tashima M., Kondo T., Ohmori K., Kurata M., Hayashi T. (2008). PD-1 PD-1 ligand interaction contributes to immunosuppressive microenvironment of Hodgkin lymphoma. Blood.

[115] Roemer M.G.M., Advani R.H., Ligon A.H., Natkunam Y., Redd R.A., Homer H., Connelly C.F., Sun H.H., Daadi S.E., Freeman G.J. (2016). PD-L1 and PD-L2 Genetic Alterations Define Classical Hodgkin Lymphoma and Predict Outcome. J. Clin. Oncol..

[116] Green M.R., Rodig S., Juszczynski P., Ouyang J., Sinha P., O’Donnell E., Neuberg D., Shipp M.A. (2012). Constitutive AP-1 activity and EBV infection induce PD-l1 in Hodgkin lymphomas and posttransplant lymphoproliferative disorders: Implications for targeted therapy. Clin. Cancer Res..

[117] Steidl C., Shah S.P., Woolcock B.W., Rui L., Kawahara M., Farinha P., Johnson N.A., Zhao Y., Telenius A., Neriah S.B. (2011). MHC class II transactivator CIITA is a recurrent gene fusion partner in lymphoid cancers. Nature.

[118] Wherry E.J. (2011). T cell exhaustion. Nat. Immunol..

[119] Śledzińska A., Menger L., Bergerhoff K., Peggs K.S., Quezada S.A. (2015). Negative immune checkpoints on T lymphocytes and their relevance to cancer immunotherapy. Mol. Oncol..

[120] Devilard E., Bertucci F., Trempat P., Bouabdallah R., Loriod B., Giaconia A., Brousset P., Granjeaud S., Nguyen C., Birnbaum D. (2002). Gene expression profiling defines molecular subtypes of classical Hodgkin’s disease. Oncogene.

[121] Sanchez-Aguilera A., Montalbán C., de la Cueva P., Sánchez-Verde L., Morente M.M., García-Cosío M., García-Laraña J., Bellas C., Provencio M., Romagosa V. (2006). Tumor microenvironment and mitotic checkpoint are key factors in the outcome of classic Hodgkin lymphoma. Blood.

[122] Scott D.W., Chan C., Hong F., Rogic S., Tan K.L., Meissner B., Ben-Neriah S., Boyle M., Kridel R., Telenius A. (2012). Gene Expression-Based Model Using Formalin-Fixed Paraffin-Embedded Biopsies Predicts Overall Survival in Advanced-Stage Classical Hodgkin Lymphoma. J. Clin. Oncol..

[123] Jachimowicz R.D., Klapper W., Glehr G., Müller H., Haverkamp H., Thorns C., Hansmann M.L., Möller P., Stein H., Rehberg T. (2021). Gene expression-based outcome prediction in advanced stage classical Hodgkin lymphoma treated with BEACOPP. Leukemia.

[124] Chan F.C., Mottok A., Gerrie A.S., Power M., Savage K.J., Shah S.P., Connors J.M., Gascoyne R.D., Scott D.W., Steidl C. (2017). Prognostic model to predict post-autologous stem-cell transplantation outcomes in classical hodgkin lymphoma. J. Clin. Oncol..

[125] Calvente L., Tremblay-LeMay R., Xu W., Chan F.C., Hong M., Zhang T., Yhim H.Y., Kuruvilla J., Crump M., Kukreti V. (2020). Validation of the RHL30 digital gene expression assay as a prognostic biomarker for relapsed Hodgkin lymphoma. Br. J. Haematol..

[126] Luminari S., Donati B., Casali M., Valli R., Santi R., Puccini B., Kovalchuk S., Ruffini A., Fama A., Berti V. (2020). A Gene Expression–based Model to Predict Metabolic Response after Two Courses of ABVD in Hodgkin Lymphoma Patients. Clin. Cancer Res..

[127] Donati B., Ferrari A., Ruffini A., Manzotti G., Fragliasso V., Merli F., Zanelli M., Valli R., Luminari S., Ciarrocchi A. (2021). Gene expression profile unveils diverse biological effect of serum vitamin D in Hodgkin’s and diffuse large B-cell lymphoma. Hematol. Oncol..

[128] Diehl V., Franklin J., Pfreundschuh M., Lathan B., Paulus U., Hasenclever D., Tesch H., Herrmann R., Dörken B., Müller-Hermelink H.-K. (2003). Standard and increased-dose BEACOPP chemotherapy compared with COPP-ABVD for advanced Hodgkin’s disease. N. Engl. J. Med..

[129] Johnson P., McKenzie H. (2015). How I treat advanced classical Hodgkin lymphoma. Blood.

[130] Duggan D.B., Petroni G.R., Johnson J.L., Glick J.H., Fisher R.I., Connors J.M., Canellos G.P., Peterson B.A. (2003). Randomized comparison of ABVD and MOPP/ABV hybrid for the treatment of advanced Hodgkin’s disease: Report of an intergroup trial. J. Clin. Oncol..

[131] Schmitz N., Pfistner B., Sextro M., Sieber M., Carella A.M., Haenel M., Boissevain F., Zschaber R., Müller P., Kirchner H. (2002). Aggressive conventional chemotherapy compared with high-dose chemotherapy with autologous haemopoietic stem-cell transplantation for relapsed chemosensitive Hodgkin’s disease: A randomised trial. Lancet.

[132] Younes A., Bartlett N.L., Leonard J.P., Kennedy D.A., Lynch C.M., Sievers E.L., Forero-Torres A. (2010). Brentuximab Vedotin (SGN-35) for Relapsed CD30-Positive Lymphomas. N. Engl. J. Med..

[133] Moskowitz A.J., Schöder H., Yahalom J., McCall S.J., Fox S.Y., Gerecitano J., Grewal R., Hamlin P.A., Horwitz S., Kobos R. (2015). PET-adapted sequential salvage therapy with brentuximab vedotin followed by augmented ifosamide, carboplatin, and etoposide for patients with relapsed and refractory Hodgkin’s lymphoma: A non-randomised, open-label, single-centre, phase 2 study. Lancet Oncol..

[134] Kumar A., Casulo C., Yahalom J., Schöder H., Barr P.M., Caron P., Chiu A., Constine L.S., Drullinsky P., Friedberg J.W. (2016). Brentuximab vedotin and AVD followed by involved-site radiotherapy in early stage, unfavorable risk Hodgkin lymphoma. Blood.

[135] Ansell S.M., Lesokhin A.M., Borrello I., Halwani A., Scott E.C., Gutierrez M., Schuster S.J., Millenson M.M., Cattry D., Freeman G.J. (2015). PD-1 Blockade with Nivolumab in Relapsed or Refractory Hodgkin’s Lymphoma. N. Engl. J. Med..

[136] Younes A., Santoro A., Shipp M., Zinzani P.L., Timmerman J.M., Ansell S., Armand P., Fanale M., Ratanatharathorn V., Kuruvilla J. (2016). Nivolumab for classical Hodgkin’s lymphoma after failure of both autologous stem-cell transplantation and brentuximab vedotin: A multicentre, multicohort, single-arm phase 2 trial. Lancet Oncol..

[137] Armand P., Shipp M.A., Ribrag V., Michot J.M., Zinzani P.L., Kuruvilla J., Snyder E.S., Ricart A.D., Balakumaran A., Rose S. (2016). Programmed death-1 blockade with pembrolizumab in patients with classical hodgkin lymphoma after brentuximab vedotin failure. J. Clin. Oncol..

[138] Moskowitz C.H., Zinzani P.L., Fanale M.A., Armand P., Johnson N.A., Radford J.A., Ribrag V., Molin D., Vassilakopoulos T.P., Tomita A. (2016). Pembrolizumab in Relapsed/Refractory Classical Hodgkin Lymphoma: Primary End Point Analysis of the Phase 2 Keynote-087 Study. Blood.

[139] Chen R., Zinzani P.L., Fanale M.A., Armand P., Johnson N.A., Brice P., Radford J., Ribrag V., Molin D., Vassilakopoulos T.P. (2017). Phase II Study of the Efficacy and Safety of Pembrolizumab for Relapsed/Refractory Classic Hodgkin Lymphoma. J. Clin. Oncol..

[140] Armand P., Kuruvilla J., Michot J.-M., Ribrag V., Zinzani P.L., Zhu Y., Marinello P., Nahar A., Moskowitz C.H. (2020). KEYNOTE-013 4-year follow-up of pembrolizumab in classical Hodgkin lymphoma after brentuximab vedotin failure. Blood Adv..

[141] Chen R., Zinzani P.L., Lee H.J., Armand P., Johnson N.A., Brice P., Radford J., Ribrag V., Molin D., Vassilakopoulos T.P. (2019). Pembrolizumab in Relapsed or Refractory Hodgkin Lymphoma: 2-Year Follow-Up of KEYNOTE-087. Blood.

[142] Postow M.A., Callahan M.K., Wolchok J.D. (2015). Immune Checkpoint Blockade in Cancer Therapy. J. Clin. Oncol..

[143] Krummel M.F., Allison J.P. (1995). CD28 and CTLA-4 have opposing effects on the response of T cells to stimulation. J. Exp. Med..

[144] Greene J.L., Leytze G.M., Emswiler J., Peach R., Bajorath J., Cosand W., Linsley P.S. (1996). Covalent dimerization of CD28/CTLA-4 and oligomerization of CD80/CD86 regulate T cell costimulatory interactions. J. Biol. Chem..

[145] Quandt D., Hoff H., Rudolph M., Fillatreau S., Brunner-Weinzierl M.C. (2007). A new role of CTLA-4 on B cells in thymus-dependent immune responses in vivo. J. Immunol..

[146] Bashey A., Medina B., Corringham S., Pasek M., Carrier E., Vrooman L., Lowy I., Solomon S.R., Morris L.E., Holland H.K. (2009). CTLA4 blockade with ipilimumab to treat relapse of malignancy after allogeneic hematopoietic cell transplantation. Blood.

[147] Diefenbach C.S., Connors J.M., Friedberg J.W., Leonard J.P., Kahl B.S., Little R.F., Baizer L., Evens A.M., Hoppe R.T., Kelly K.M. (2017). Hodgkin lymphoma: Current status and clinical trial recommendations. J. Natl. Cancer Inst..

[148] Mottok A., Steidl C. (2018). Biology of classical Hodgkin lymphoma: Implications for prognosis and novel therapies. Blood.

[149] Lamprecht B., Walter K., Kreher S., Kumar R., Hummel M., Lenze D., Köchert K., Bouhlel M.A., Richter J., Soler E. (2010). Derepression of an endogenous long terminal repeat activates the CSF1R proto-oncogene in human lymphoma. Nat. Med..

[150] Von Tresckow B., Morschhauser F., Ribrag V., Topp M.S., Chien C., Seetharam S., Aquino R., Kotoulek S., De Boer C.J., Engert A. (2015). An Open-Label, Multicenter, Phase I/II Study of JNJ-40346527, a CSF-1R Inhibitor, in Patients with Relapsed or Refractory Hodgkin Lymphoma. Clin. Cancer Res..

[151] Hombach A., Jung W., Pohl C., Renner C., Sahin U., Schmits R., Wolf J., Kapp U., Diehl V., Pfreundschuh M. (1993). A CD16/CD30 bispecific monoclonal antibody induces lysis of hodgkin’s cells by unstimulated natural killer cells In AND In vivo. Int. J. Cancer.

[152] Rothe A., Sasse S., Topp M.S., Eichenauer D.A., Hummel H., Reiners K.S., Dietlein M., Kuhnert G., Kessler J., Buerkle C. (2015). A phase 1 study of the bispecific anti-CD30/CD16A antibody construct AFM13 in patients with relapsed or refractory Hodgkin lymphoma. Blood.

[153] Ramos C.A., Ballard B., Zhang H., Dakhova O., Gee A.P., Mei Z., Bilgi M., Wu M.F., Liu H., Grilley B. (2017). Clinical and immunological responses after CD30-specific chimeric antigen receptor-redirected lymphocytes. J. Clin. Investig..

[154] Ruella M., Klichinsky M., Kenderian S.S., Shestova O., Ziober A., Kraft D.O., Feldman M., Wasik M.A., June C.H., Gill S. (2017). Overcoming the immunosuppressive tumor microenvironment of Hodgkin lymphoma using chimeric antigen receptor T cells. Cancer Discov..

[155] Fromm J.R. (2011). Flow cytometric analysis of CD123 is useful for immunophenotyping classical Hodgkin lymphoma. Cytom. Part B Clin. Cytom..

